# Mapping capacity to conduct health technology assessment in Central, Eastern and South-Eastern Europe

**DOI:** 10.3325/cmj.2016.57.66

**Published:** 2016-02

**Authors:** Antonio Olry de Labry Lima, Leticia García Mochón, Araceli Caro Martínez, Eva Martín Ruiz, Jaime Espín Balbino

**Affiliations:** 1 Andalusian School of Public Health (EASP), Campus Universitario de Cartuja. Granada, Spain; 2 CIBER en Epidemiología y Salud Pública (CIBERESP), Spain; 3 Instituto de Investigación Biosanitaria ibs. Granada. Hospitales Universitarios de Granada/Universidad de Granada, Granada, Spain

## Abstract

**Aim:**

To provide insights into the capacity to conduct health technology assessment (HTA) in Central, Eastern, and South-Eastern Europe (CESEE), taking account of technical, financial, networking, and human resources.

**Methods:**

An e-mail survey of 257 CESEE key informants involved in HTA was undertaken between March and April 2014. Contact e-mail addresses were identified from the internet. The survey questionnaire consisted of 3 sections: i) characteristics of the organization performing HTA, (ii) networking in HTA, and (iii) resources allocated for HTA.

**Results:**

The survey was completed by 41 respondents representing a wide range of institutions from CESEE countries (response rate of 19.8%). Less than a quarter of respondents reported that their institutions had HTA-specific budgets, whereas the majority indicated that their institutions participated in HTA networks either at domestic or international levels. Although almost half of respondents indicated that their institutions offered HTA training, a shortage in skills training was suggested as the main barrier to HTA.

**Conclusion:**

This is the first survey to thoroughly assess the state of HTA capacity in the CESEE region. To strengthen HTA capacity, CESEE countries should increase financial, technical, and training resources. To strengthen collaboration, the European Union and other international bodies should assist existing HTA networks in fulfilling their regional activities through leadership, advocacy to local policymakers, funding, and technical assistance.

Health technology assessment (HTA) is a multidisciplinary process that uses multiple approaches and techniques to provide comprehensive, objective information for health policymakers to make decisions on public access to and funding for products, services, and programs. Effective decision-making should include multiple criteria (eg, medical, economic, ethical, social, legal, cultural), thus requiring multi-disciplinary teams of experts working together to produce assessments (1).

HTA methods and processes are increasingly used all around the world (2). In countries with mature systems in place (eg, Western Europe, North America), HTA products are integrated into policy, governance, reimbursement, and/or regulation. By contrast, countries with greater budget and human resource limitations lack capacity to fulfil their HTA needs. These constraints lead policymakers and payers to consider HTA products produced in other settings as an aid to decision-making at domestic level. However, there are critical factors that hamper the transferability of HTA products across jurisdictions, including unit costs, resource utilization, and unmet medical needs. In addition, decision-makers do not generally have the necessary skills to interpret and use HTA products, often resulting in decisions made intuitively or focused exclusively on budget impact considerations (3-6).

Although a number of publications have looked into the capacity to undertake HTA in Western European settings, no paper has thoroughly explored this issue in Central, Eastern, and South Eastern European (CESEE) countries as yet (4,7,8). Taking into consideration the problems faced by low- and middle-income countries, the European Commission's Seventh Framework Programme (FP7) ADVANCE_HTA project has among its aims to improve the implementation and capacity building of HTA in emerging settings (9). Hence, one of its sub-projects involves the mapping of current capacities to perform HTA in emerging settings of CESEE, not only including decision-making bodies but also other institutions.

This article reports the findings from an international cross-sectional survey of organizations involved with HTA processes or decision-making. Specifically, the survey aims to provide insights into the capacity to conduct HTA of the CESEE countries, taking account of technical, financial, networking, and human resources.

## Materials and methods

A cross-sectional survey was performed in March-April 2014 targeting people who worked at CESEE institutions potentially related with decision-making bodies or HTA processes, such as ministries of health, hospitals, universities, and public health agencies. E-mail addresses of potential survey respondents were identified by searching the databases of the following online resources: World Health Organization Regional Office for Europe (10), International Society for Pharmacoeconomics and Outcomes Research (ISPOR) Regional Chapters (11), Forum for Public Health in South Eastern Europe (12), Association of Schools of Public Health in the European Region (ASPHER) (13), European Health Technology Assessment Network (14), European University Association (15), and the author information section of PubMed search.

A survey questionnaire was devised by following an iterative process. A draft was developed based on previous HTA capacity surveys undertaken by RedETSA (16) (HTA Network of the Americas) and EUnetHTA (European Network for Health Technology Assessment) in 2008 (17). The questionnaire was pilot tested among regional HTA experts and revised several times following the feedback received. The questionnaire consisted of three sections: (i) characteristics of the organization performing HTA, (ii) networking in HTA, and (iii) resources allocated for HTA, with 23 questions. The time needed to fulfill the questionnaire was about 5-10 minutes.

All potential respondents were contacted via e-mail with a cover letter including a brief explanation of the survey, and assured of confidentiality. No ethical approval was needed, according to the project technical proposal, due to that we were not dealing with patient information data. To maximize response rates four reminders were sent.

## Results

A total of 257 subjects were identified and approached with a survey. A total of 41 responses were received, generating a response rate of 19.8%. A great heterogeneity in respondent characteristics (eg, institution type, responsibility, HTA experience) was observed. Most replies were collected from Lithuania (5, 12.2% of all questionnaires received), followed by Romania and Slovenia with 4 replies each (9.76%), and Bulgaria, Greece, and Hungary with 3 replies each (7.32%). Montenegro, Kosovo, and Bosnia and Herzegovina offered no responses. Two respondents (both from Cyprus) rejected to answer the questionnaire because they felt unqualified. Thus, 39 replies were usable, with 29 respondents (74.4%) reporting that their organizations were capable to perform HTA and 2 of them (5.1%) being unaware of their institutions' HTA activities.

A total of 8 respondents (20.5%) indicated that their institutions had HTA-specific budgets. The main lines of work reported were heath policy (19, 65.5%) and HTA (18, 62.1%), followed by health care organization and management (15, 51.7%) and research (14, 48.3%). The main HTA product types produced were systematic reviews, economic evaluations, and technical reports/working documents (15, 51.7% each). Concerning the scope of collaboration with other institutions, the majority of respondents reported having partnerships with domestic (government agency, 79.3%; academia/university, 62.1%; professional association, 58.6%) and international institutions (government agency, 58.6%; academia/university, 44.8%) (Supplementary Table 1(web extra material 1)).

When asked about bibliographic databases being used at institutional level, respondents cited MEDLINE/PubMed (96.6%), The Cochrane Library (79.3%), and Embase (65.5%) as the most common. Almost half of respondents (12, 41.4%) indicated that their institutions offered HTA training to external participants, while well over half of respondents (19, 65.5%) reported having strategies for dissemination of HTA products. Requests to perform HTA were generally received from governments (18, 62.1%) and private companies (9, 31%).

Finally, the most important limitation faced by institutions involved in HTA was funding limitation (n = 24), followed by lack of skills training (n = 21). Lack of access to network was mentioned by 11 respondents (4 respondents stated domestic network and 7 international network) and the lack of institutional support was stated by 5 respondents (Supplementary Table 1(web extra material 1)). With respect to barriers to conducting HTA encountered by institutions not engaged in the practice, the most cited limitations were of political nature (Table 1).

**Table 1:** Types of limitations faced by institutions not performing health technology assessment*

Limitation type	Yes, n (%)
Lack of funding	2 (28.6)
Lack of time	0 (0)
Lack of human resources	1 (14.3)
Lack of access to network	1 (14.3)
Lack of institutional support	1 (14.3)
No interest	0 (0)
Others^†^	5 (71.5)

*Multiple choice answer.

†Lack of political support, no invitation from authorities and solely in health technology assessment agency.

## Discussion

This survey represents the first systematic attempt to mapping HTA capacity in the CESEE region. The ultimate goal of this study is to inform the development of a toolbox for emerging countries containing best practices and recommendations on HTA and decision-making. The results of the present study suggest that there are several important areas that require attention so as to strengthen the capacity of CESEE countries to undertake HTA.

First, although almost all survey respondents reported using MEDLINE/PubMed, the use of complementary databases appears to be less common. This finding is worth mentioning because using just one bibliographic database is considered inadequate. For instance, the overlap in journals covered by MEDLINE/PubMed and Embase is estimated to be just 34% (18). As a result, we recommend the involvement of librarians in HTA since it has shown to strengthen the capacity to produce HTAs (19).

Second, consistent with findings by the EunetHTA project on mature HTA systems, the main limitations to undertaking HTA were lack of funding and training opportunities. The remarkable position occupied by the shortage of training initiatives has important implications, because the surveyed institutions mostly engage in high impact activities such as health policy and HTA. Deficiencies in knowledge and skills in HTA producers may lead policymakers to disregard the findings of HTA products, which ultimately would have a negative impact on health care quality (20,21). As the Cox report (22) suggests, by maximizing the potential of HTA, decision-makers will be better able to implement decisions that capture the benefits of new technologies, overcome uncertainties, and recognize the value of innovation. Furthermore, well-conducted HTA require multidisciplinary teams and adaptive human resources. This highlights the need for training programs to ensure that trained personnel, representing different disciplines but having a common language, can cooperate in performing HTAs (8). For these reasons, we recommend to increase funds for HTA, with emphasis on capacity building.

Lastly, as revealed by the survey results, HTA is no longer conducted in isolation. An increased and closer collaboration at both national and international levels could help overcome the difficulties reported in the survey. Cooperation among HTA organizations appears to be extremely relevant for countries without institutionalized HTA, because it offers the opportunity to learn from others’ experiences and benefit from HTA studies that have already been completed elsewhere. However, caution should be exercised, as the transferability of HTA evidence across settings is not always straightforward. Although both European Union (EU) countries and non-EU countries have a high level of collaboration in the field of HTA, more often than not, only a small proportion of an HTA institution’s activities are embedded in international projects. Hence, the EU should promote and support transnational collaborations in HTA, because it can help reduce duplication, thereby enabling resources to be used more efficiently and assuring the quality and timeliness of HTA products.

It is plausible that a number of limitations may have influenced the obtained results. The first is the relatively low response rate; survey participation is a particularly acute issue for web surveys, which tend to suffer from lower response rates (10%-25%) than other survey modes (23). We found that this may be due to language barriers or contacted individuals considering themselves unqualified to respond. Although attempts were made to increase the number of responses by sending reminders, this strategy did not render significant improvements. Nonetheless, the survey response rate echoes those obtained in similar studies related to HTA (8,24-27). The second is the representativeness of the responses, since it is possible that respondents answered on an individual rather than official basis (ie, not gathering a response from their institutions). These limitations underline the difficulty of collecting data on HTA from emerging settings, such as those within the CESEE region. Lastly, this low response rate could reflect the poor situation with HTA in these countries.

The findings of this international survey provide insights into the level of capacity in HTA of organizations from the CESEE region. Of particular note, the findings suggest a need for increased technical, financial, and training support, which could be fulfilled by reinforcing existing HTA networks and with additional support from international bodies, such as the EU.
