# Supplementary material for: Mapping capacity to conduct health technology assessment in Central, Eastern and South-Eastern Europe
Source: Croat Med J. 2016 Feb;57(1):66–70. doi: 10.3325/cmj.2016.57.66 (PMC4800330; doi:10.3325/cmj.2016.57.66)
Supplement: Supplementary Table 1 [file CroatMedJ_57_s001.pdf]

Supplementary material 1. Main characteristics of institutions performing health technology assessment (n=29)††

Domain		Yes, n (%)
Main line of work	Health technology assessment (HTA)	18 (62.1%)
	Funding of research	4 (13.8%)
	Clinical practice guidelines	7 (24.1%)
	Conducting research	14 (48.3%)
	Quality assessment and patient safety	12 (41.4%)
	Healthcare organisation and management	15 (51.7%)
	Heath policy activities	19 (65.5%)
	Other*	4 (10.1%)
Scope of collaboration with others institutions	Domestic	11 (39.9%)
	International	1 (3.6%)
	Both domestic and international	16 (57.1%)
Type of HTA product	Systematic review	15 (38.5%)
	Economic evaluation	15 (38.5%)
	Clinical practice guideline	5 (12.8%)
	Technical report/working document	15 (38.5%)
	Other†	6 (15.6%)
Type of institutional partner at domestic level (n=28)	Government agency	23 (79.3%)
	Hospital	15 (51.7%)
	Academia/university	18 (62.1%)
	Professional association	17 (58.6%)
	Industry	16 (55.2%)
	Others§	3 (10.2%)
Type of institutional partner at international level (n=27)	Government agency	17 (58.6%)
	Hospital	6 (20.7%)
	Academia/university	13 (44.8%)
	Professional association	7 (24.1%)
	Industry	5 (17.2%)
	Others¶	4 (14.6%)
Type of requester of HTA product	Government	18 (62.1%)
	Health professional	4 (13.8%)
	Private company	9 (31.0%)
	Consumers/patients/users	5 (17.2%)
	Other	7 (23.8%)
Type of limitation faced by the institution	Skills training	21 (72.4%)
	Domestic HTA network	4 (13.8%)
	International HTA network	7 (24.1%)
	Institutional support	5 (17.2%)
	Other**	5 (17.2%)

*Accreditation, classic public health functions, health information, e-health and providing compulsory health insurance

†Administrative act decision, rational pharmacotherapy guidelines within reimbursement system, critical appraisal, critical review of health economics and rapid relative effectiveness assessment

§Medical and nursing chambers, non-governmental organisation, public health centres

¶European Network for Health Technology Assessment (EUnetHTA), Piperska Group, World Health Organization, United Nations International Children's Emergency Fund (UNICEF), etc.

**Access to databases, human resources, lack of more extensive support by clients, lack of real-life outcome data, legal framework, and insufficient number of staff.

†† Multiple choice answer. When all the responses was not achieved, the number of responses are specified.
